# An electromyographic and kinematic study of the scapular stabilisers

**DOI:** 10.4102/sajp.v76i1.1413

**Published:** 2020-09-30

**Authors:** Sonia Briel, Benita Olivier, Witness Mudzi

**Affiliations:** 1 Department of Physiotherapy, Faculty of Health Sciences, University of the Witwatersrand, Johannesburg, South Africa; 2 Postgraduate School, University of the Free State, Bloemfontein, South Africa

**Keywords:** EMG activity, serratus anterior lower fibres, lower trapezius, scapular, kinematics

## Abstract

**Background:**

The scapular stabilisers, especially the actions of the force couples around the scapula, have an impact on the biomechanics of the scapula and the orientation of the glenoid.

**Objectives:**

The aim of our study was to determine both the muscle activity and the correlation between the muscle activity ratio of the lower force couple (the serratus anterior lower fibres and the lower trapezius).

**Methods:**

This was a quantitative cross-sectional study. Muscle activity of the dominant serratus anterior lower fibres and the lower trapezius muscles was collected with surface electromyographic (EMG) sensors and an inertial motion capture system was used to measure the three-dimensional (3D) shoulder flexion in the sagittal plane and abduction in the frontal plane. Graph Pad 5 (Prism, San Diego, CA, USA) was used for the statistical analysis. The confidence level was set at 95% (*p* < 0.05).

**Results:**

Sixteen men and women participated in our study, with a mean (standard deviation) age of 25.4 (± 4.6) years, weight of 80.2 (± 25.1) kg and height of 171.6 (± 10.3) cm. A strong negative correlation was found at the start of the abduction (*r* = −0.623; *p* = 0.01) between the muscle activity of the serratus anterior lower fibres and the lower trapezius.

**Conclusions:**

The only significant increase in the mean EMG ratio of serratus anterior lower fibres versus the lower trapezius was present at 60% (from baseline) of abduction (*p* = 0.03).

**Clinical implications:**

The EMG activity ratio of serratus anterior lower fibres and lower trapezius remains variable in different movement planes.

## Introduction

There is conflicting evidence on the electromyographic (EMG) recruitment patterns of the scapular stabilisers in the literature. Historically, Inman was the first researcher to study and analyse the scapulothoracic movements in 1944. He examined shoulder elevation in the coronal plane in asymptomatic subjects (Struyf et al. 2014). The serratus anterior is thought to be more active in forward flexion (Inman et al. 1944). Later work by Wadsworth and Bullock-Saxton (1997) found that the upper trapezius was recruited first, followed by the middle trapezius and then the lower trapezius. Some researchers agreed that all parts of the trapezius were more active in abduction than in flexion (Bagg & Forrest 1986; Inman et al. 1944). However, more recent research has concluded that serratus anterior lower fibres are activated more in abduction and scaption (abduction in the scapular plane) (Ludewig & Reynolds 2009; McClure et al. 2001; Smith et al. 2003). Other researchers agree that all parts of the trapezius are more active in abduction than in flexion (Ludewig & Reynolds 2009; McClure et al. 2001).

Furthermore, imbalances in the EMG ratios of the lower trapezius, serratus anterior lower fibres and middle trapezius have been found in injured population groups (Cools et al. 2007; Karduna et al. 2005; Myers et al. 2005). The alignment of the scapula on the thorax is dependent on the ideal length, tension and recruitment of the scapular stabilising muscles. This allows the correct positioning of the glenoid for articulation at the glenohumeral joint during the elevation of the humerus (Neuman 2010).

The coordinated kinematics of the scapula and the humerus around the thorax during arm elevation in the sagittal, scapular and/or frontal planes is essential for full, pain-free glenohumeral articulation (Parel et al. 2012). It was concluded by Bdaiwi et al. (2015) that, during simultaneous or individual neuromuscular stimulation of the serratus anterior and the lower trapezius, an increase in the acromiohumeral distance was observed. Holterman et al. (2009) used EMG biofeedback to monitor the serratus anterior lower fibres and the lower trapezius and observed that during specific activation of the serratus anterior lower fibres, spontaneous synergistic activation of the lower trapezius occurred. The serratus anterior lower fibres and the lower trapezius act as a synergistic force couple. This agrees with the findings of Inman et al. (1944), who argued that the lower force couple of the scapula might consist of the serratus anterior and the lower trapezius. Inman et al. (1944) conceptualised the concept of force couples, in particular, the upper force couple consisting of serratus anterior upper fibres and the upper trapezius, and the lower force couple consisting of the serratus anterior lower fibres and the lower trapezius. Smith et al. (2003) drew attention to the fact that, anatomically, the upper trapezius is more involved in the elevation of the scapula and that only the lower fibres of the serratus anterior and the lower trapezius form the lower force couple. It is thus well acknowledged in the literature that the actions of the scapular stabilisers, especially the actions of the force couples around the scapula, have an impact on the positions of the scapula and the glenoid.

The classical description of scapulohumeral movement or rhythm, where the range (0º – 180°) is divided into three distinct phases, is still valid and applicable today (Bagg & Forrest 1988; Inman et al. 1944). In scapulohumeral movement or rhythm, the range (0º – 180°) is divided into three distinct phases (Bagg & Forrest 1988; Inman et al. 1944). The first setting phase is 0º – 30° (in abduction) and 0º – 60° (in flexion) (Bagg & Forrest 1988; Inman et al. 1944). Most of the movement during this phase takes place at the glenohumeral joint (Inman et al. 1944). During the middle phase (81.8º – 139.1°), the movement is mostly at the scapulothoracic joint (Bagg & Forrest 1988). In the final phase (140º – 180°), most of the movement takes place at the glenohumeral joint (Bagg & Forrest 1988). Scapular kinematics has been used successfully by numerous researchers in the study of the movement of the scapula in normal shoulders (Bonnefoy-Mazure et al. 2010; Šenk & Chĕze 2006) and pathological shoulders (Ludewig & Cook 2000; Rundquist et al. 2003).

The objectives of our study were to determine the muscle activity of the lower trapezius and the serratus anterior lower fibres, in the movement of forward flexion in the sagittal plane and abduction in the frontal plane. The correlation between the EMG activity ratios of the lower force couple – serratus anterior lower fibres and the lower trapezius – was also determined. If specific EMG activity ratios exist in healthy shoulders, the findings can be applied during the rehabilitation process to painful and pathological shoulders.

## Method

This was a descriptive quantitative cross-sectional study conducted in the movement analysis laboratory of the Physiotherapy Department at the University of the Witwatersrand.

### Study population and sampling strategy

Asymptomatic adults were recruited from the student body of the University of the Witwatersrand, schools, church groups and sports clubs. The age range of the participants was 18–35 years. Pathology is less likely to occur in this age group, hence the inclusion of this specific age group (Pribicevic 2012). Participants with healthy shoulders, without any shoulder pain or surgery and without any cervical pain or surgery (determined through a pre-testing questionnaire), were recruited. A convenience sample was used. A small sample was included in the EMG and kinematic data collection; the numbers used in EMG and kinematic studies are frequently smaller because of the complex nature of the collection and analysis processes (Forte et al. 2009; Wattanaprakornhul & Halakim 2011).

### Data collection

Data collection took place from 27 June 2016 to 18 March 2017. In the familiarisation sessions, all the participants read and completed the informed consent forms, were weighed, measured and kinematic inertial measurements were collected and recorded.

Muscle activity of the dominant serratus anterior lower fibres and the lower trapezius muscles was collected using the eight-sensor Trigno wireless set (Delsys, Inc., Natick, MA, USA). The skin was cleaned with a commercially available paste, Nuprep (Weaver and Company, Aurora, CO, USA), to reduce skin impedance prior to the EMG electrodes being applied to the skin (skin impedance typically < 10 kΩ) (Konrad 2005). Tensospray (BSN Medical GmbH, Quickbornstrasse, Hamburg, Germany), for improved adherence of the electrodes, was applied prior to the electrodes being attached. The muscles were tested and normalisation with maximum isometric voluntary contraction (MVIC) was done.

**FIGURE 1:**
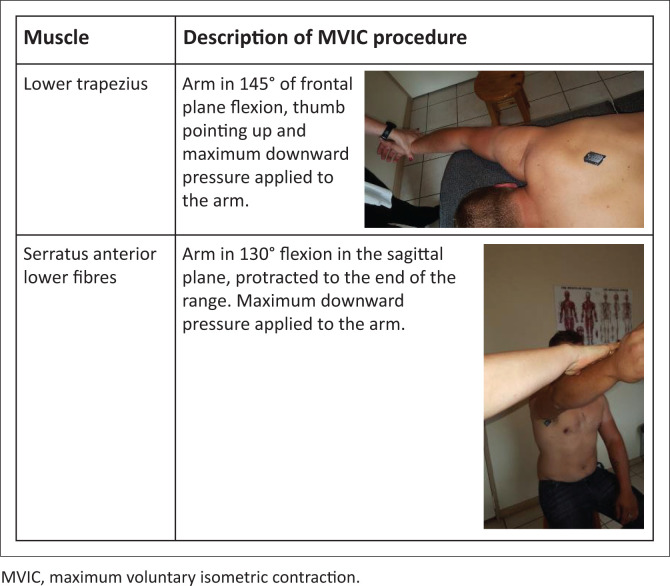
Muscle description and testing positions.

Testing positions of the lower trapezius and serratus anterior lower fibres are shown in Figure 1. Participants performed a 3 second isometric contraction (for a count of 1001, 1002, 1003) (MVIC) against maximum manual resistance applied by the first author. A 2 min pause (timed with a digital watch) occurred between muscle contractions (Ivey et al. 1985). The muscle activity was recorded at every 10% of the cycle, from the start (neutral position = 0%) to the maximum angle (end of range = 100%). The participants were tested in the positions as described by Hislop and Montgomery (1995) for the lower trapezius and serratus anterior lower fibres.

The Xsens MVN-Link Biomech (Xsense, 2016 [Xsens Technologies B.V., Enschede, the Netherlands]) inertial motion capture system was used to measure the three-dimensional (3D) shoulder flexion in the sagittal plane and abduction in the frontal plane. The participant wore an Xsens bodysuit. The inertial sensors were placed on predetermined anatomical sites as recommended by Xsens Technologies B.V. These were the sternum, spine of the scapula, humerus, hand, pelvis, upper leg, lower leg and foot. The Xsens system was calibrated using sensor-to-segment calibration, which defines the anatomical coordinate system of the thorax, scapula and humerus and relates them to the technical coordinate system of the corresponding MTx sensors (Cutti et al. 2010).

On completion of the calibration, the kinematic and EMG movement testing was done. Electromyographic and kinematic signals were triggered to occur simultaneously with the Trigno EMG trigger sensor at the start, after calibration had occurred and before the actual movements took place. This was done to ensure that EMG and kinematic data were collected simultaneously. With the results obtained from the kinematic data, the flexion and abduction angles were recorded at every 1% of the movement cycle from the start (or neutral position) to the maximum angle (or end of the range).

The movement of flexion in the sagittal plane was repeated and recorded, slowly and steadily, three times, to a verbal count of five for the up and a count of five for the down movement. The abduction movement was measured in the frontal plane and this movement was also repeated three times, also slowly and steadily, for a verbal count of five for the up and a count of five for the down movement.

### Data reduction

The Euler rotational sequence of XZY for abduction in the frontal plane of movement and XYZ for the flexion in the sagittal plane of movement was used to analyse the joint angles. Flexion and abduction angles were recorded every 1% of the movement cycle from the start, or neutral position, to the maximum angle, or what we consider clinically as the full range. By reporting the joint angles at different percentages, comparisons could be drawn between the EMGs (%MVIC) of the two muscles under investigation (serratus anterior lower fibres and the lower trapezius) at specific joint angles. For example, the specific %MVIC of serratus anterior lower fibres could be determined at 60% of the movement cycle. A comparison could then be made to the specific %MVIC of the lower trapezius at the exact same joint angle of 60%. The muscle activity was calculated as a percentage of the MVIC of the serratus anterior lower fibres and the lower trapezius.

From the kinematic data, the flexion and abduction angles were recorded at every 1% of the movement cycle, from the start (or neutral position) to the maximum angle (or end of the range). The muscle activity was recorded at every 10% of the cycle, from the start (neutral position = 0%) to the maximum angle (end of range = 100%). Graphs were used to allow conversion from percentages range of movement (ROM) (0% – 100%) to degrees ROM (0º – 180°) to be made. The range was represented in percentages on the horizontal axis (0% – 100%) and in degrees on the vertical axis (0° – 180°). For example, when the abduction graph was used, 60% of movement on the horizontal axis correlates with 110° on the vertical axis. The same example applied to the flexion graph used. This conversion from percentages to degrees makes comparisons to present studies easier.

### Statistical analysis

Graph Pad 5 (Prism, San Diego, CA, USA) was used for the statistical analysis. The confidence level (CL) was set at 95% (*p* < 0.05). The Shapiro–Wilk’s test was used to determine the normality of the data. It was concluded that the majority of the data were not normally distributed. For the relationship of the ratio of serratus anterior lower fibres and the lower trapezius in the two movement planes of flexion and abduction, Spearman’s correlation was employed.

### Ethical consideration

Ethical clearance was obtained from the Human Research Ethics Committee (Medical) of the University of the Witwatersrand (clearance number: M160515, 27/06/2016) before the commencement of our study. Participants were informed about our study prior to their participation. Written informed consent to take part in the study was obtained from all the participants before data collection took place. Written informed consent to perform the video recordings was also provided by all the participants, who took part in the video recording during the kinematic sessions before the actual video recording took place.

## Results

Sixteen (eight women and eight men) participants took part in the study. All participants were right-handed and the right arm of all the participants was used for the collection of the kinematic data, in flexion and in abduction.

All the participants between 18 and 35 years of age (Table 1) were recruited from the University of the Witwatersrand and the general population. Inclusion criteria were set to include anyone without current or previous shoulder pain, dysfunction or cervical pain and dysfunction for 3 months before our study. Patients with a history of shoulder or cervical surgery were excluded.

**TABLE 1:** Demographic and anthropometric information of the participants (*n* = 16).

Variables	Combined group	Females	Males
Age (years)	25.4 ± 4.6	24.9 ± 4.7	25.9 ± 4.7
Mass (kg)	80.2 ± 25.1	69.0 ± 11.9	91.1 ± 29.6
Height (cm)	171.6 ± 10.3	165.0 ± 6.6	178.0 ± 9.3

A comparison of the EMG ratios of serratus anterior lower fibres and the lower trapezius during flexion in the sagittal plane of movement and abduction in the frontal plane of movement is shown in Table 2. The only significant increase in the mean EMG ratio of serratus anterior lower fibres versus the lower trapezius in the movements of sagittal flexion versus frontal abduction was present at 60% (from baseline) of frontal abduction (*p* = 0.03).

**TABLE 2:** A comparison between the electromyographic mean muscle activity ratios (*n* = 16).

Range	Flexion		Abduction		*p*
	Mean	s.d	Mean	s.d.	
Neutral	3.37	2.57	3.60	5.61	0.82
10%	3.21	3.26	2.64	2.46	0.29
20%	3.59	4.62	2.98	4.90	0.42
30%	3.08	3.52	3.17	6.72	0.93
40%	2.96	3.75	3.20	6.47	0.84
50%	3.81	4.56	2.07	3.87	0.8
60%	3.52	4.32	1.76	2.76	0.03
70%	3.74	2.93	1.61	2.86	0.07
80%	3.23	0.79	1.97	3.58	0.10
90%	2.70	0.70	2.41	5.21	0.69
Max	2.40	0.59	2.62	5.14	0.64

s.d., standard deviation.

Table 3 shows the correlation between the EMG ratios of serratus anterior lower fibres and the lower trapezius in flexion and abduction. For the correlation between EMG (%MVIC) of serratus anterior lower fibres and the lower trapezius in abduction, there was a strong negative correlation at the start of abduction (*p* = 0.01; *r* = −0.623) and at 10% of abduction (*p* = 0.004; *r* = −0.675). No correlation existed between the EMG (%MVIC) ratio of serratus anterior lower fibres and the lower trapezius in flexion at the start (*p* > 0.05; *r* = −0.061) or at 10% (*p* > 0.05; *r* = −0.211) of the movement. For the remainder of the movement cycle, to full ROM, of both fllexion and abduction, a poor correlation existed between the EMG (%MVIC) ratios of serratus anterior lower fibres and the lower trapezius (*p* > 0.05). The ratios remained variable between the serratus anterior lower fibres and the lower trapezius for the rest of the movement cycle from 20% to 100%, in both flexion and abduction.

**TABLE 3:** The correlation between the electromyographic ratios of serratus anterior lower fibres and the lower trapezius in flexion and abduction (*n* = 16).

Range	Flexion		Abduction	
	*r*	*p*	*r*	*p*
Start	−0.061	0.823	−0.623	0.010
10%	−0.211	0.433	−0.675	0.004
20%	−0.333	0.208	−0.476	0.062
30%	−0.181	0.502	−0.300	0.258
40%	0.082	0.763	−0.186	0.491
50%	0.059	0.827	−0.238	0.375
60%	−0.006	0.983	−0.184	0.494
70%	−0.131	0.629	−0.162	0.549
80%	−0.147	0.587	−0.079	0.771
90%	−0.394	0.131	−0.098	0.717
Max	−0.282	0.289	−0.166	0.537

## Discussion

The results yielded increased activity for the serratus anterior lower fibres in the higher ranges of movement, in both sagittal flexion and frontal abduction. These results were supported by Ekstrom, Donatelli and Soderberg (2003), who concluded that the maximum activity in serratus anterior lower fibres was reached in arm elevation above 120° in various planes. In scapulohumeral movement, the range (0º – 180°) is divided into distinct phases (scapulohumeral rhythm). Three phases have been identified (Bagg & Forrest 1988; Inman et al. 1944). The first setting phase is 0º – 30° (in abduction) and 0º – 60° (in flexion) (Bagg & Forrest 1988; Inman et al. 1944). Most of the movement during this phase takes place at the glenohumeral joint (Inman et al. 1944). During the middle phase (81.8º – 139.1°), the movement is mostly at the scapulothoracic joint (Bagg & Forrest 1988). In the final phase (140º – 180°), most of the movement takes place at the glenohumeral joint (Bagg & Forrest 1988).

The serratus anterior lower fibres and the lower trapezius are seen by many as the only true upward rotators of the scapula (Ekstrom, Bifulco & Lopau 2004; Phadke, Camargo & Ludewig 2009). The upward rotation of the scapula by the serratus anterior lower fibres is counteracted by the synchronous activity of the lower trapezius (Perry 1978). Converting the range of movement from percentages to degrees in our study allows the following conclusions to be drawn. In our study for glenohumeral abduction, an increase in the EMG activity in serratus anterior lower fibres was noted from 60% to 80%, which correlates to 100º – 140° or mid-range of the movement cycle. This is similar to the results obtained by Ludewig and Cook (2000), who found an increase in the serratus anterior muscle EMG activity from 61º to 120° (mid-range) of glenohumeral abduction. In a study by Wickham et al. (2010), conducted in the frontal abduction plane from 120º to 135°, serratus anterior fibres reached 85% (%MVIC) of muscle activity compared to the lower trapezius, which reached 80% (%MVIC) of muscle activity. Similar results were achieved in our study, which indicates higher serratus anterior lower fibre muscle EMG activity versus the lower trapezius in frontal abduction. Our results show that at 80% – 90% (120° – 160°) of the movement cycle in abduction in the frontal plane, the serratus anterior lower fibres reached 80% of the MVIC compared to the lower trapezius, which reached 50% of MVIC. The clinical significance of the concluded higher levels of muscle activity present in serratus anterior lower fibres during the frontal abduction movement lies in the clinical application thereof. Specific attention should be given to the strengthening of the serratus anterior lower fibres during the scapular rehabilitation phase. The balanced action of the synergistic force couple of serratus anterior lower fibres and the lower trapezius should therefore theoretically provide controlled upward rotation of the scapula during the abduction movement. Improved scapular control could hence lead to less biomechanical impingement during glenohumeral movement.

A major finding in our study was the increased EMG activity of the serratus anterior lower fibres compared with the lower trapezius in the 70% – 90% (120º – 160°) of the frontal abduction movement. Wadsworth and Bullock-Saxton (1997) observed conflicting evidence of the recruitment of the serratus anterior lower fibres and the lower trapezius in flexion and abduction. Moseley et al. (1992) found that the EMG activity of the serratus anterior lower part progressively increased during the active elevation of the scapula, in the plane of the scapula (30° anterior to the frontal plane) and the serratus anterior was also considered to be more active in forward flexion (Inman et al. 1944). The movements in the study by Inman et al. (1944) were also conducted in the plane of the scapula. There were similarities between our study and those by Decker et al. (1999) and Cools et al. (2007). The participants were similar: the mean age was the same, between 20.7 and 30.4 years; the sample sizes were between 20 and 45 participants; both studies focused on normal shoulders; and in both studies the conclusion reached was that serratus anterior was active mostly in scaption (30° anterior to the coronal plane). The increased activity of serratus anterior lower fibres in our study found in frontal abduction and by previous researchers in the plane of the scapula, might be because of the small difference in the movement planes. Scaption is abduction in the plane of the scapula, that is 30° anterior to the coronal or frontal plane. Pure abduction, on the other hand, is in the coronal or frontal plane. The findings that the serratus anterior lower fibres are more active in the abduction plane, regardless of the movement being in pure abduction in the coronal plane or abduction in 30° anterior to the frontal plane (scaption), might thus support rather than contradict each other. These results were supported by Ekstrom et al. (2003), who concluded that the maximum activity in serratus anterior lower fibres was reached in arm elevation above 120° in various planes. Decreased range of glenohumeral movement observed during clinical examination in either the pure frontal abduction or the scapular (scaption) plane of movement could potentially point to the underlying weakness of the serratus anterior lower fibres.

The increased activity of the serratus anterior lower fibres displayed at 70% – 90% (120º – 160°) of the movement cycle in abduction might be explained by the fact that posterior tilt of the scapula occurs near the end of the range of abduction in the frontal plane. Serratus anterior lower fibres are the main muscle component involved in the posterior tilt action of the scapula (Ekstrom et al. 2004). External rotation (or posterior tilt) of the scapula occurs at the end of the range of humeral elevation (Ludewig & Reynolds 2009; McClure et al. 2001). The finding of increased muscle activity of the serratus anterior in the frontal plane of abduction in the higher ranges (70% – 90%; 120º – 160º ) of the movement cycle is therefore supported by the findings of the authors who are mentioned above.

Our results demonstrated greater muscle activity of the serratus anterior lower fibres and the lower trapezius as the movement cycle increased, in both flexion in the sagittal plane and abduction in the frontal plane of movement. The muscle activity of the serratus anterior lower fibres and the lower trapezius was similar during flexion. Significantly more activity of the serratus anterior lower fibres was found in frontal abduction. There was an increased EMG activity of the serratus anterior lower fibres and the lower trapezius in the mid-range, from 60% to 90% (100° – 140°), of the movement cycle. The concluded findings of our study are similar to the results of Bagg and Forrest (1988), who found that during the middle phase of abduction (81.8º – 139.1°), the ratio of scapulothoracic to glenohumeral rotation was 1.71° to 0.71°. An explanation of the higher scapular to glenohumeral rotation, offered by Freedman and Munro (1966) and Doody, Freedman and Waterland (1970), was that the moment arms of the scapular rotators exceeded the moment arm strength of the supraspinatus and the deltoid in this range of movement. The scapulothoracic movement of upward rotation of the scapula is mainly executed by the serratus anterior lower fibres and the lower trapezius. The synergistic force couple of the serratus anterior lower fibres and the lower trapezius is of particular importance to control the upward rotation of the scapula in the higher ranges of movement. The importance of this finding can apply to clinical practice. Patients presenting with subacromial impingement frequently experience pain in the mid-range (80° – 140°) of the shoulder abduction movement. A contributing factor to the pain experienced in the mid-range of the abduction movement might, therefore, be the underlying weakness of the serratus anterior lower fibres. The biomechanical implication of this weakness can be seen as causative of impingement of the subacromial structures because of a lack of upward rotation of the scapula in this particular part of the range of movement.

Our results led to the conclusion that there is an increased EMG activity of serratus anterior lower fibres and the lower trapezius in the higher movement planes of abduction and flexion. It can be concluded that both the serratus anterior lower fibres and the lower trapezius are active throughout the flexion movement. Our results are similar to the findings of Wattanaprakornhul and Halakim (2011), who also concluded that both the lower trapezius and serratus anterior lower are active in flexion throughout the range of movement. Their study parameters were similar to those of ours: the participants were of a similar age range (19–47 years), the sample number was 15 and normal shoulders were studied. The importance of strengthening of the scapular upward rotators, the serratus anterior lower fibres and the lower trapezius, is hence echoed in the findings of our study and also highlighted in current literature.

The correlation between the EMG muscle activity of the lower force couple (*n* = 16), found a significant negative relationship at the start of abduction (*p* < 0.01; *r* = 0.623) and at 10% of the abduction movement (*p* < 0.05; *r* = −0.675). For the rest of the movement, the muscle activity of serratus anterior lower fibres and the lower trapezius provided no correlation (Figure 1 and Table 1). Just because the two independent variables of the serratus anterior lower fibres and the lower trapezius in the movement planes of flexion in the sagittal plane and abduction in the frontal plane bear no linear relationship, it does not mean that they are unrelated. Wickham et al. (2010) also found variable EMG muscle activity ratios. They concluded that EMG muscle activity of the scapular stabilisers remains variable in the middle and higher ranges of movement in flexion and abduction.

## Conclusion

It was determined that the serratus anterior lower fibres were significantly more active than the lower trapezius in the frontal plane of abduction. No correlation exists, except for a strong negative correlation from the start to 10% of movement between the serratus anterior lower fibres and the lower trapezius in abduction in the frontal plane of movement. The relationship between the two variables, however, showed a consistent increase in serratus anterior lower fibres muscle activity in the frontal abduction plane.

## Clinical implications

No specific ratio correlation was expressed between serratus anterior lower fibres and the lower trapezius in the movement planes of flexion in the sagittal plane and abduction in the frontal plane. Regardless of this finding, the serratus anterior lower fibres demonstrated greater EMG activity in the higher ranges (120º – 180°) of the abduction movement in the frontal plane of movement.

## Limitations

The sample size used in this study provided enough power for the inferential statistics used. Using larger sample sizes from healthy individuals can add to the database of our study. Reference can be made to patients between 18 to 35 years of age. This can, however, not be applied directly to the general population.
